# Measuring the effectiveness of career education at a medical university and future issues from the perspective of students’ transformation: impact of a new career education program at a medical university on entrepreneurship effectiveness

**DOI:** 10.20407/fmj.2024-014

**Published:** 2024-10-31

**Authors:** Shuichi Murakawa, Junko Ishiguro, Akiko Watanabe

**Affiliations:** 1 Center for Society-Academia Collaboration, Fujita Health University, Toyoake, Aichi, Japan; 2 Faculty of Business Administration, Toyo Gakuen University, Bunkyo-ku, Tokyo, Japan; 3 School of Medical Sciences, Fujita Health University, Toyoake, Aichi, Japan

**Keywords:** Career design, Career education, Medical school, Entrepreneurship, Essential competencies

## Abstract

**Objectives:**

The current study aimed to examine the effectiveness of a new career education course, “Career Development Theory,” at a medical university. This course is designed to enable students to plan their careers and consider various career options. This class was introduced to improve students’ entrepreneurial qualities and broaden their career choices.

**Methods:**

The research methods included a survey to measure entrepreneurial qualities before and after attending the class and an analysis of students’ reports of what they had learned from the class. A total of 243 students who plan to qualify to participate in the national examinations for clinical technologists, clinical engineering technologists, and radiology technologists participated in the research.

**Results:**

The results revealed that autonomy and creativity scores significantly increased, whereas risk-taking scores decreased. This suggests that the class may have taught students accurate self- and risk-assessment skills while fostering a cautious attitude toward avoiding unnecessary risks. The report analysis indicated that students’ self-awareness and attitudes toward their careers changed because of the class. Many students felt that their career options had expanded and found avenues outside the medical profession.

**Conclusions:**

This research demonstrates the need for and effectiveness of career education in medical universities and provides valuable suggestions for improving educational programs. The future goal is for more students to design their careers freely and choose the path that best suits them from various career options. The current results may provide a useful reference for career education curriculum development at other medical universities.

## Introduction

A special characteristic of medical universities is that choosing a major is directly related to students’ career choices. In Japan, most students have already made post-graduation career choices when they consider entering university. After entering university, students typically set their university study goals to pass the national examination and obtain entry into their chosen professions.

In a previous study, approximately half of high school students who had decided to enter a medical university expressed views similar to the following sentiment: “I vaguely thought that I wanted to work in a hospital or the medical profession. During my secondary education, I learned about a specific profession, became convinced that it was the profession I desired, and decided to pursue it”.^1^ The report suggested that 50% of the students enrolled in medical universities choose their future occupations for vague reasons.^1^

In international studies, it has been reported that medical students’ career intentions and preferences change both at the time of admission and graduation, depending on various factors.^2^ Furthermore, the medical career decision-making process was reported to still be controversial in a previous literature review on medical career decision-making.^3^ In addition, a study examining a new generation of medical students (Generation Z), including third- and fourth-year high school students, who were aspiring to study human medicine in Northern Switzerland, reported that students were interested in studying human medicine as a discipline, as well as for helping and healing people, and that they were motivated to study it continuously.^4^ A systematic review of research examining the motivation of medical students to select medical studies reported that the main motivating factors were interest in medicine and science, work identification, job and financial security, and humanitarian service.^5^

In Japan, the number of training schools for medical professionals is increasing annually, highlighting the need to improve the educational system so that graduating students can find employment in medical facilities and related fields.^6^ In addition, even for holders of national qualifications that are difficult to obtain, the necessity to strengthen career support at higher education institutions has been emphasized as opportunities to use these qualifications have become more diverse.^1,5,7^ To promote reforms in the way doctors work, moves to encourage task shifting/sharing among medical professionals are being promoted.^8^ One type of human resources required in the era of rapid changes in volatility, uncertainty, complexity, and ambiguity (VUCA) is basic skills for working adults, as advocated by the Ministry of Economy, Trade, and Industry.^9^

However, the postgraduate career paths of medical students are diverse. According to a survey by the Graduate School of Health Sciences, the School of Health Sciences and Medical Sciences at Fujita Health University (FHU), the number of medical students employed outside hospitals was three out of 414 (0.7%) in 2012, while the number was 29 out of 582 (5.0%) in 2022, showing an increasing trend. According to a survey conducted by the Ministry of Health, Labour, and Welfare on job turnover among new hires who graduated in March 2020, the job turnover rates within 3 years of employment in the medical and welfare industry were 38.8% for university graduates (up 0.2 points from the previous year) and 46.4% for high school graduates (similar to the rate for all industries in Japan). This is higher than the turnover rates for new hires in all sectors in Japan (32.3% for college graduates and 37.0% for high school graduates). College and high school graduates are prevalent in the top five industries with the highest turnover rates in the medical and welfare sectors.^10^

Once considered a stable career choice based on qualifications, medical qualification holders are losing stability because of technological innovations, systemic changes, and increasing job mobility. Against this backdrop, one program provides career education from a long-term perspective, even in medical universities that have not traditionally emphasized career education. Career education is “education that promotes career development through the cultivation of abilities and attitudes that form the necessary foundation for individual social and occupational independence.”^11^ In 2003, the Japanese government created the “Youth Independence and Challenge Plan,” leading to a full-fledged advocacy of “career education.”^12^ However, medical universities, in which qualifications and employment have traditionally been directly linked, have lagged in their career education efforts. Despite the changes described above, we have moved toward introducing a new curriculum. The importance of leadership in graduate medical education has been recognized, and curriculum development is encouraged.^13^

The current study examined the effectiveness of the new career education class introduced in the first-year education program at FHU by analyzing survey results of students’ entrepreneurial qualities before and after the class, and investigating student reports about their learning. The results of the current study may be helpful for examining the content and methods of the new class.

### Class outline

The subject of this study was a newly established “Career Development” class at FHU, which began in 2022. This class is required (one credit, 30 hours) in the first semester of the first year of enrollment in the basic field. The class was conducted from April to July 2022, with the following objectives for students:

1.  Know yourself and draw a career map.
2.  Develop a proactive attitude toward life.
3.  Learn and acquire the elements necessary to work as a team.

The contents of all 12 lectures are listed in Table 1. Five external lecturers, including those with medical backgrounds, were invited to teach the course.

The first half of each 90-minute class session consisted of a lecture with assignments that were based on themes related to the lecture content and workbook, followed by group work and presentations in the last 40 minutes. Groups of 5–6 students worked on the assigned themes related to lecture content in the first half, then shared their learning with the whole class. Therefore, the class was not limited to passive knowledge acquisition and included group work on issues with no correct answers.

## Methods

The research was conducted in the following three steps:

We analyzed the “Survey of Entrepreneurial Qualities” in students who took the Career Development Theory course (Research 1), and the reports submitted as “Learning in Career Development Theory” after all 12 classes (Research 2). Subsequently, the student reports in which scores changed significantly from Research 1 to Research 2 were analyzed and typified (Research 3).

### Participants

The participants in this study were 243 students who were enrolled in the Career Development class offered at FHU in the first semester of FY2022. These students plan to qualify national examinations for clinical, clinical engineering, and radiology technologists upon graduation.

### Method of investigation

#### Research 1

The authors conducted the survey using The General Measure of Enterprising Tendency Test (GET2),^14^ at the start (April 2022) and at the end (July 2022) of the class, and clarified changes in the students at the two time points. The GET2 measures the Need for Achievement (NACH) using a 12-item, two-component method, autonomy (AUTO) using a six-item, two-component method, and measures creativity (CT), risk-taking (RT) and locus of control (LOC) using 12-item, two-component methods. A total of 54 questions were included in the survey. This class had the following achievement objectives: “to acquire a proactive attitude” and “to acquire flexible thinking and to think about one’s way of life and life planning.” This test was adopted because of its high compatibility with these achievement objectives.

Simultaneously, we asked participants about their entrepreneurial readiness (two items, two-case method). In recent years, substantial attention has focused on fostering an entrepreneurial approach among students. Entrepreneurship can be defined as “the spirit to take action beyond the framework and create new value.”^15^ In this class, sufficient time is set aside for group work and class-wide presentations. Students listen to other opinions and learn more about their values and perspectives. Such opportunities are considered to broaden students’ perspectives on career choices and encourage proactive planning. Therefore, we measured entrepreneurial thinking in the current study.

##### Analysis method

Correspondence t-tests were conducted using Excel to determine whether taking the Career Development Theory class impacted entrepreneurial readiness. The entrepreneurial readiness ratio was calculated using Ishiguro and Ohe’s (2019)^16^ method. The situation in which both self-confidence and motivation are present is shown as “Ready” (hereafter abbreviated by the initial letter “R”). Self-confidence is abbreviated as “R” (this is considered the same as the previous situation), “Dormant” refers to individuals who are confident but not motivated, “Potential” refers to individuals who are motivated but not confident, and “No No” refers to individuals lacking any of the three (Table 2).

#### Research 2

We asked 243 students to submit reports on their learning in the 2022 Career Development class. Because the reports were the subject matter of the class evaluations, after being finalized, the personal information of 229 students who consented to participate was deleted and replaced with an irregular number. Subsequently, a random number table was used to select 30 reports for analysis.

We carefully read the contents of the reports and extracted sentences describing “what I learned from the career development class” and “the impact of the career development class on the students.” The semantic content of the statements was captured and categorized on the basis of their similarities and differences. Two researchers conducted the analysis, and the results were repeatedly reviewed to ensure credibility, confirmability, and clarity.

#### Research 3

Students whose scores (on a 100-point scale) changed by +10 or more (absolute values) from the data obtained from the GET2 measurements in Research 1 were examined. We selected five students whose attitudes toward entrepreneurship changed positively regarding motivation and confidence. We re-read the reports and searched for key sentences regarding the influences that changed their attitudes and behaviors, such as which points in the lectures attracted and influenced them. We extracted and categorized the sentences regarding the “elements and factors.” In addition, the sentences related to “results/changes” (e.g., whether a particular point in the course led to changes in one’s behavior) were extracted and categorized.

### Ethical considerations

In conducting this research, we attended a research ethics seminar at our institution. We complied with the “Declaration of Helsinki” and the “Ethical Guidelines for Life Sciences and Medical Research Involving Human Subjects.” Special consideration was given to the following points in Studies 1–3:

#### Research 1

The purpose of the cooperation request, the response method, and the survey method were explained, and voluntary cooperation in the research was verbally requested in class. Students’ agreement to cooperate in the research was confirmed via a question in Google Forms. It was explained verbally and in writing at the beginning of the Google Form that the presence or absence of research cooperation would not affect or disadvantage the student’s academic performance, that the student could withdraw consent up to the point at which the analysis was started by separating personal information, and that withdrawal of consent would not affect or disadvantage the student’s academic performance.

To clarify the changes between the two time points, the responses in Google Forms required the input of student ID numbers and names to identify individuals. One researcher was in charge of data analysis, and the data were given to them in a form that separated personally identifiable information from the responses of only participants who agreed to take part in the research, with encrypted numbers allowing for before-and-after comparisons. The contrast tables were stored separately from the electronic data.

#### Research 2

As the reports to be studied were subject to evaluation in class, we confirmed students’ willingness to cooperate in online research when the class evaluations were finalized. We explained that cooperation was voluntary and unconnected to class evaluations, and that the research results would be published in academic papers and presented at conferences, while maintaining confidentiality.

Because the reports were uploaded to the learning management system and downloaded in a personally identifiable manner, on completion of the evaluation, personal information was removed and replaced with an irregular number. The 30 reports to be analyzed were then extracted using a random number table.

#### Research 3

A data analyst encrypted the reports of the five participants, and the data manager (principal investigator) collected the reports of the corresponding participants and shared them among the data analysts in Research 2.

## Results

### Research 1: Results of a survey of entrepreneurial qualities of medical students

The results of 161 (66.2%) respondents who responded to both surveys at the two time points and whose consent to participate in the research was confirmed were included in the analysis.

**Figure 1:**
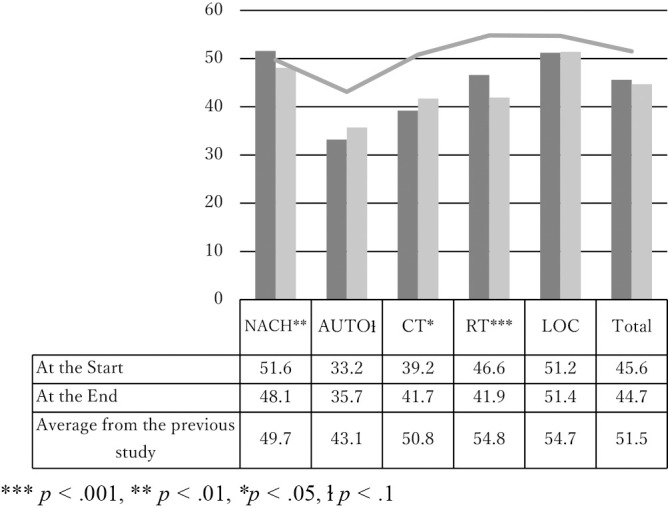
GET2 Scores (each item converted to 100 points)

Figure 1 shows the starting and ending scores for this class. For comparison, a survey conducted by Ishiguro and Ohe (2019) on 224 Japanese students (not including medical students) is included as an average.

By item, the desire for achievement (NACH) and risk-taking (RT) scores significantly decreased from 51.6 to 48.1 and 46.6 to 41.9, respectively. However, the autonomy (AUTO) and creativity scores increased significantly from 33.2 to 35.7 and 39.2 to 41.7, respectively. Compared with the scores of Japanese university students reported by Ishiguro and Ohe (2019), all scores in the present research were lower, except for the need for achievement at the beginning. In particular, the RT score at the end of the current study was more than 10 points lower than that of the Japanese students examined by Ishiguro and Ohe (2019).

Table 3 shows the state of participants’ entrepreneurial awareness at the start and end of the program. Approximately 90% of respondents were neither confident nor motivated to start businesses at the beginning of the survey. Even at the end of the program, 85.1% of participants were neither confident nor motivated, and were categorized as “No No”.

**Figure 2:**
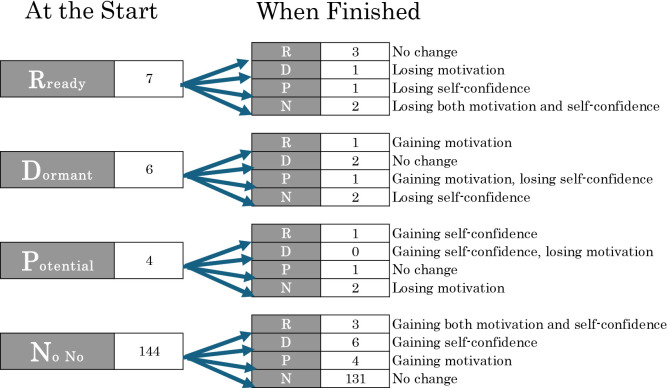
Change in attitude toward entrepreneurship (persons)

Figure 2 shows changes in the views of participants in each category at the end of the program. After the program, 137 out of 161 participants (85.1%) belonged to the same category and showed no change. Nine participants gained motivation to start a business, and 10 gained confidence to start a business. However, five participants lost motivation, and six lost confidence. Some exhibited positive changes toward entrepreneurship, whereas others exhibited negative changes regarding entrepreneurship. In all cases, it appears that even short-term activities changed participants’ perceptions through exposure to actual entrepreneurial activity.

### Research 2

The following six categories of learning were identified for students who took the Career Development class: (1) changes in how students perceive the subject matter, (2) how to work and build a career, (3) expansion of career options after graduation, (4) importance of affirming and accepting oneself, (5) importance of learning and accepting differences in values, and (6) clarification of issues to be addressed. These six categories were extracted.

Some students stated that they did not understand the need to study Career Development before the class began, but that the lectures by outside instructors changed their perception. Additionally, by receiving lectures from professionals and people with entrepreneurial experience with whom they had no previous contact, the students learned about ways to work outside hospitals even after obtaining national certification; they also learned “how to work and build a career” by becoming aware of the career they wanted to pursue after learning about various ways of working, as well as learning about occupations and ways of working that were previously unknown to them. They also felt that they could expand their career options by learning about occupations and working methods they had not previously known about. In addition, by using the textbook “Self-Design Book” and the exchange of opinions in group work, the students learned the importance of affirming and accepting themselves, knowing that people have various values, and the importance of building relationships with others (Table 4).

### Research 3: Trends and results extracted from assignment reports of students with significant changes in Research 1

The elements/factors that attracted or influenced the students in the lecture were classified into four categories: (1) experiences of entrepreneurs in the medical field, (2) opportunities to face and understand oneself, (3) opportunities to think about future society and the self, and (4) group work/discussion. The results were categorized into the following four areas: (1) broadening of horizons and knowledge about a variety of careers, (2) acquiring a positive attitude about living in one’s own way and increasing self-confidence, (3) clarifying future goals and easing anxiety about the future, and (4) realizing the importance of diverse values and communication.

**Figure 3:**
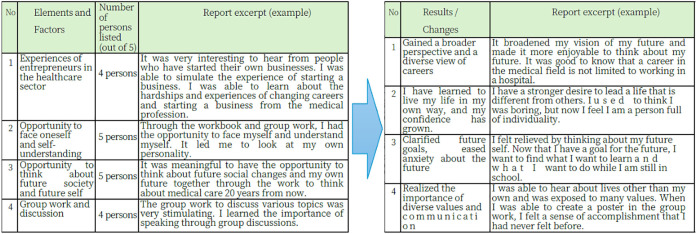
Analysis and extraction of top 5 reports with large score changes in GET2

The following section describes each category. Descriptions extracted from participants’ responses are shown in *italics in parentheses*. 1. Entrepreneurial experiences in the medical field demonstrated the significance of learning about various careers through the opportunity to hear real stories of entrepreneurs. The lectures also discussed the effectiveness of the entrepreneurial experience as a simulation of career choices. In the lecture, the speaker stated that learning about diverse careers from multiple entrepreneurs had a positive impact. 2. The second section discussed “Opportunities to face oneself and develop self-understanding,” the importance of knowing oneself and facing oneself. Regarding section 2, the importance of knowing and facing oneself was described by one participant as follows: “*Through the workbook and group work, I had the opportunity to face and understand myself. This led me to examine my personality.*” Participants also stated that the workbook and group work allowed them to examine themselves and consider their characteristics. In addition, one participant stated “*I became more determined to lead a different life from others. I used to think I was boring, but now I feel I am full of individuality.*” Positive statements included, “I have come to feel full of individuality.” 3. In the “Opportunities to think about future society and my future self” section, participants described a possible future for society and their vision for it. In section 3, the lecturers discussed the future of society and their future vision: “It *was meaningful to have an opportunity to think about future social changes and my own future through the work of thinking about medical care 20 years from now. I learned that some people start their own companies, even in the medical profession, and that I have a wide range *of* choices. I had not considered careers other than working in a hospital after graduation.*” 4. Group work and discussion: “*I enjoyed the stimulating group work in which everyone discussed various topics. I learned about the importance of talking to others through group discussions. I heard about the lives of other people and was exposed to a range of values. I felt a sense of accomplishment that I had never felt before when I made a poster as part of the group work.*” These comments illustrate the effectiveness of the teaching methods that incorporated group work and discussion (Figure 3).

## Discussion

### 1. The impact of changes in GET2 scores on entrepreneurial qualities such as autonomy

The “autonomy” and “creativity” scores increased after the Career Development class. As indicated in the class outline, this resulted from the time spent in group work on tasks with no correct answers, as well as passive knowledge acquisition. Over 15 years have passed since the necessity of first-year education and active learning at universities was first advocated for, and there have been many reports on project-based learning (PBL) class development.^17,18^ A meta-cognitive scale report indicated that PBL methods increase analytical ability, address issues, adjust goals, revise plans, and change strategies on the basis of what is grasped.^18^ The results of Ibuki et al. appear to be similar to the increase in the score of “autonomy” revealed in the current results. Regarding international studies, the results of a systematic review examining the role of the PBL approach in teaching and learning physics revealed positive effects of PBL on improving academic achievement, attitudes to learning physics, problem-solving, critical and creative thinking abilities, and cooperative learning enhancement.^19^ Furthermore, a review of new and emerging educational technologies in problem-based curricula, with a specific focus on three cognate clinical disciplines (medicine, dentistry, and speech and hearing sciences) reported a generally positive effect of the adoption of various educational technologies in PBL.^20^ Additionally, the rise of massive online open courses in all fields, including health sciences, has been proposed to be effective, particularly for continuous medical education and public health literacy.^20^

The Vision for Future Talent (Ministry of Economy, Trade and Industry, 2022) described the demand for skills and abilities required by presidents and executives of large companies competing in the global market. According to the report, “attentiveness/no mistakes,” “responsibility/sincerity,” and “trustworthiness/sincerity” were the top-ranked skills in the 2015 survey. However, in 2050, they top-ranked skills were predicted to change to “problem-finding ability,” “accurate forecasting,” and “innovative ability.” In the current study, students’ scores in “autonomy” and “creativity” increased, suggesting that the Career Development class may contribute to improving abilities required in society in the future.

### 2. The impact of the class format on students’ career choices

Some students expressed that their career options expanded after taking the course, as shown in Table 4. Many medical university students have already made future career choices when they enter university. Research on former students who dropped out of the Department of Rehabilitation reported that academic failure, university maladjustment, and financial reasons were the most common reasons for dropping out. Additionally, the most common reason for withdrawal by academic year was university maladjustment during the second year.^21^ In a survey conducted by the Ministry of Education, Culture, Sports, Science and Technology (MEXT) on the status of students dropping out of school and taking a leave of absence,^22^ the most commonly reported reasons were economic hardship, poor academic performance, and employment. An increased percentage of students dropping out of school because of poor academic performance has been noted. Kitamura et al. (2019) reported that 43% of students answered, “I can now do what I want to do” and “I am doing what I want to do” after leaving school, suggesting that students did not fully consider their vocational understanding and purpose of higher education before choosing a career path. In the current study, among medical students who reported the “expansion of career choices,” the class may have influenced the future self-image of individuals who had decided on a school of higher education on the basis of a vague image of their occupation. Research 3 showed that listening to the diverse views and life experiences of professionals and businesspeople in the guest lectures in this class influenced students’ career choices.

### 3. Risk-taking score

As shown in the results of Research 1, risk-taking scores decreased by approximately 5 points on the GET2 after the class. The students may have heard about the obstacles and failures entrepreneurs faced, which may have made them think, “I will try not to be like them,” and “I will avoid risk if I sense the possibility of risk.” However, it has been reported that successful entrepreneurs choose their risks carefully,^23^ and that they seek to undertake challenges but do not gamble.^24^ Thus, successful entrepreneurs tend to assess a situation, understand their capabilities, and decide whether they can cope. Yamakawa^25^ points out that Japanese people tend to view failure as taboo and do not tell others about it. In contrast, in the United States, there are multiple terms for failure (e.g., failure, loss, mistake) and many individuals value and talk about what they learned from their failures and how they recovered from them. Yamakawa^25^ also reported that failure does not negate the individual, but requires awareness of the need to prevent its recurrence. Thus, regarding our lectures on Career Development, the results suggest that it will be necessary to create an environment with a sense of learning together from failures and risks, and to create an environment in which students can recognize failures rather than avoiding risks in future classes.

## Conclusion

The current study aimed to clarify the effects of a newly introduced career education class at a medical university by examining changes in the results of a survey of students’ entrepreneurial qualities before and after the class, and an analysis of reports submitted by students on their learning in the class. Regarding the acquisition of basic skills for working adults, which is the purpose of introducing career education, the measured scores of “autonomy” and “creativity” increased in terms of independence and imagination, showing a certain level of effectiveness. In addition, while aiming to broaden the perspectives of medical students by exposing them to diverse perspectives about the lives of many professionals and entrepreneurs, the results suggested that the program led to behavioral change, such as students’ proactive attitudes and clarification of behavioral goals, as well as broadening students’ perspectives through group work by exposing them to diverse ways of thinking. In addition, analysis of students’ reports revealed behavioral changes, such as students’ proactive approach and clarification of behavioral goals. The results of the analysis and the verification of the effectiveness of the class suggest that the current findings may be useful for developing class content and methods for career education classes in future.

## Figures and Tables

**Table1:** Career development course content and basic skills for adult workers to be acquired in 2022

	Lesson content		Lesson content
1	Introduction Student self-introduction	7	Career always starts “now” (**ability to execute**).
2	Communication (**ability to communicate**, **ability to listen**)	8	Draw up a career plan that is unique to you (**planning skills**)
3	Think about the purpose of coming to the university (look back, face yourself)	9	Clarification of issues necessary for oneself (**independence**, **problem-finding ability**)
4	Envisioning the ideal university life (self-actualization, **creativity**)	10	Thinking and creating on your own (**ability to work**, **flexibility**, **creativity**)
5	Knowing one’s own identity and working on one’s own initiative **(independence)**	11	Group work (ability to **communicate**, **listening**, **flexibility**, **situational awareness**, **discipline**, **stress control**)
6	Considering specific plans for self-actualization (self-actualization, **planning skills**)	12	Group work presentation (ability to **communicate**, **ability to listen**, **flexibility**)

Note: Sessions 5–9 were requested from outside lecturers.

**Table2:** Entrepreneurial awareness framework

		“Are you confident that you can become an entrepreneur?”	
		Yes	No
“Do you want to launch a new business?”	Yes	Ready	Potential
		I want to be an entrepreneur and I’m confident I can be one.	I want to be an entrepreneur, but I don’t think I can be one.
	No	Dormant	No No
		I can be an entrepreneur, but I don’t want to be one.	I don’t think I want to be or can be an entrepreneur

**Table3:** Change in attitude toward entrepreneurship

	At the start	At the end
R	4.3%	5.0%
D	3.7%	5.6%
P	2.5%	4.3%
N	89.4%	85.1%

**Table4:** Learning by attending the career development theory lectures

Category	Excerpt from report content (example)
Changes in acceptance and perception	I had always wanted to work in a hospital, so before taking the Career Development class, I wondered why it was a required course. I thought a career was still a long way off, and that it was of little relevance to me going into the medical field.
How to work and build a career	Until I took this class, I thought that all clinical technologists worked in hospitals or laboratories. However, as I took the class and listened to the stories of various people, I learned that this was not the case. Although I did not change my conclusion, I wanted to work in a hospital after learning that hospitals are not the only place I could work. The class gave me a chance to think about what I want to do and how I want to live my life.
Expanding career options	The class broadened my perspective on my future and gave me a variety of options. I learned that hospitals are not the only places where medical professionals are active. I learned that taking 4 years to find myself can broaden my future career options.
The importance of self-affirmation and acceptance	I thought it was important to analyze myself and how to change to positive thinking. ... (omission) ... I think that thinking about how to make use of those weaknesses and how to make use of the good points will also be important in choosing a future career. I thought it was important not to deny that I am not good enough, but to find the good points and have confidence that I can do this better than others. I have the disadvantage of caring too much about other people, but I have found that I am good at thinking about the feelings of people in many directions and following up to prevent people from feeling uncomfortable. I consider this to be part of my personality.
The importance of knowing and accepting differences in values	I realized once again that friends are a big part of enriching one’s life, and that they are indispensable. I will be involved with many people in the future, and I want to build good relationships with the people I meet. To build a relationship with people from different backgrounds, backgrounds, and values, it is important not to deny the other person’s perspective, not to imagine the person in one’s own image, and to accept the other person.
Clarification of issues to be addressed	I would like to pay attention to various things so that I can connect them to my future. I strongly felt that it would be a waste to spend 4 years of university life just doing nothing and then realize that it was over. I have learned that you don’t have to have a big dream, but if you want to achieve a goal, you can start by taking some action, even if it is small. On this basis, I thought about a career plan for my university life. The first step is to acquire knowledge. Next is to develop my strengths. I will then be able to provide high quality medical care in the future.
